# Smartphone- and Tablet-Based Tools to Assess Cognition in Individuals With Preclinical Alzheimer Disease and Mild Cognitive Impairment: Scoping Review

**DOI:** 10.2196/65297

**Published:** 2025-05-27

**Authors:** Rosanne L van den Berg, Sophie M van der Landen, Matthijs J Keijzer, Aniek M van Gils, Maureen van Dam, Kirsten A Ziesemer, Roos J Jutten, John E Harrison, Casper de Boer, Wiesje M van der Flier, Sietske AM Sikkes

**Affiliations:** 1 Alzheimer Center Amsterdam Neurology Amsterdam University Medical Center Amsterdam The Netherlands; 2 Neurodegeneration Amsterdam Neuroscience Amsterdam The Netherlands; 3 Department of Clinical, Neuro and Developmental Psychology Faculty of Movement and Behavioral Sciences VU University Amsterdam The Netherlands; 4 Neurocast BV Amsterdam The Netherlands; 5 Medical Library VU University Amsterdam The Netherlands; 6 Metis Cognition Ltd. Kilmington Common United Kingdom; 7 Department of Psychiatry, Psychology & Neuroscience King's College London London United Kingdom; 8 Department of Epidemiology and Biostatistics Amsterdam Neuroscience VU University Medical Center Amsterdam The Netherlands

**Keywords:** preclinical Alzheimer disease, mild cognitive impairment, cognition, smartphone-based assessment, tablet-based assessment, digital biomarkers, digital health, psychometrics, scoping review

## Abstract

**Background:**

Assessment of cognitive decline in the earliest stages of Alzheimer disease (AD) is important but challenging. AD is a neurodegenerative disease characterized by gradual cognitive decline. Disease stages range from preclinical AD, in which individuals are cognitively unimpaired, to mild cognitive impairment (MCI) and dementia. Digital technologies promise to enable detection of early, subtle cognitive changes. Although the field of digital cognitive biomarkers is rapidly evolving, a comprehensive overview of the reporting of psychometric properties (ie, validity, reliability, responsiveness, and clinical meaningfulness) is missing. Insight into the extent to which these properties are evaluated is needed to identify the validation steps toward implementation.

**Objective:**

This scoping review aimed to identify the reporting on quality characteristics of smartphone- and tablet-based cognitive tools with potential for remote administration in individuals with preclinical AD or MCI. We focused on both psychometric properties and practical tool characteristics.

**Methods:**

This scoping review was conducted following the PRISMA-ScR (Preferred Reporting Items for Systematic Reviews and Meta-Analyses extension for Scoping Reviews) guidelines. In total, 4 databases (PubMed, Embase, Web of Science, and PsycINFO) were systematically searched from January 1, 2008, to January 5, 2023. Studies were included that assessed the psychometric properties of cognitive smartphone- or tablet-based tools with potential for remote administration in individuals with preclinical AD or MCI. In total, 2 reviewers independently screened titles and abstracts in ASReview, a screening tool that combines manual and automatic screening using an active learning algorithm. Thereafter, we manually screened full texts in the web application Rayyan. For each included study, 2 reviewers independently explored the reported information on practical and psychometric properties. For each psychometric property, examples were provided narratively.

**Results:**

In total, 11,300 deduplicated studies were identified in the search. After screening, 50 studies describing 37 different digital tools were included in this review. Average administration time was 13.8 (SD 10.1; range 1-32) minutes, but for 38% (14/37) of the tools, this was not described. Most tools (31/37, 84%) were examined in 1 language. The investigated populations were mainly individuals with MCI (34/37, 92%), and fewer tools were examined in individuals with preclinical AD (8/37, 22%). For almost all tools (36/37, 97%), construct validity was assessed through evaluation of clinical or biological associations or relevant group differences. For a small number of tools, information on structural validity (3/37, 8%), test-retest reliability (12/37, 32%), responsiveness (6/37, 16%), or clinical meaningfulness (0%) was reported.

**Conclusions:**

Numerous smartphone- and tablet-based tools to assess cognition in early AD are being developed, whereas studies concerning their psychometric properties are limited. Often, initial validation steps have been taken, yet further validation and careful selection of psychometrically valid outcome scores are required to demonstrate clinical usefulness with regard to the context of use, which is essential for implementation.

## Introduction

### Background

Alzheimer disease (AD) is a neurodegenerative disease associated with gradual decline in cognition, where disease stages range from preclinical AD, in which cognitive decline is absent or only subtle, to mild cognitive impairment (MCI) and dementia [1,2]. Biologically, the disease is defined by pathological changes in amyloid accumulation and neurofibrillary tau protein tangles that appear years before the onset of cognitive symptoms [1-3]. However, it has been widely shown that, even in the preclinical stage, cognition does decline subtly [4-7]. With the increasing number of older adults, it is expected that AD will become a major societal problem, estimated to affect 131 million people in 2050 worldwide [8,9]. The emergence of AD biomarkers allows for the recognition of the disease in the preclinical stage, which has opened a window of opportunity for treatment studies, as interventions are likely to be most beneficial in early stages of the disease. Accordingly, disease-modifying treatments and nonpharmaceutical prevention trials have increasingly focused on preclinical stages of AD over the past years [10-12]. This shift toward the earliest AD stages highlights the need for new tools that provide outcome measures of cognition that are adequate for use in these early disease stages in the realm of early detection, diagnosis, disease monitoring, and evaluation of treatment effects [13-17]. In addition, to enable large-scale decentralized prevention, intervention, or disease-monitoring initiatives, tools are required that enable remote, time-efficient, and reliable assessment of cognition.

The current gold standard to assess cognition in AD is through neuropsychological testing, using paper-and-pencil tests that need to be supervised by a trained neuropsychologist. Importantly, such traditional cognitive tests are not suitable for the earliest AD stages as these tests often lack the sensitivity that is required to detect subtle cognitive changes [7,17,18]. Thus, novel, stage-specific testing paradigms are needed, and digital tools are promising to fill this gap [19,20]. A major advantage of digital cognitive tools is their suitability for unsupervised remote assessment, which enables highly frequent testing that may provide a more accurate reflection of cognition [21,22]. Given the intuitive person-device interaction of touch screen devices, smartphone- and tablet-based tools provide the optimal modality for unsupervised remote assessment [23,24]. Other advantages of such cognitive tools include reduced patient burden; time efficiency through automatic administration and scoring; high scalability; and the potential for rich data collection of precise measurements, including, for example, response times.

Over the last decade, numerous digital tools have been developed that have the potential for remote assessment of cognition in early AD stages [19]. However, the development of such tools has not yet led to large-scale implementation [25]. To date, the measurement quality of digital tools to assess cognition is largely unknown, although it has widely been acknowledged that information on psychometric properties is important for the use and implementation of these tools [19,20,26-28]. For the evaluation of cognitive tests in early AD stages, a recommendation framework has been proposed based on the Consensus-Based Standards for the Selection of Health Measurement Instruments methodology [16,29,30]. This framework highlights the importance of psychometric properties concerning structural and construct validity, responsiveness, and clinical meaningfulness. Hence, available information on psychometric properties is a prerequisite for the successful implementation of digital tools. Although previous reviews have described some validation aspects of digital cognitive assessments [19,31], a comprehensive overview of the reporting of crucial psychometric properties (ie, structural and construct validity, reliability, responsiveness, and clinical meaningfulness) of digital cognitive tools for use in preclinical AD or MCI is currently missing.

### Objectives

This scoping review was conducted to identify the reporting on quality characteristics of smartphone- and tablet-based tools to assess cognition in individuals with preclinical AD or MCI. Our scope was limited to smartphone- and tablet-based cognitive tools with the potential for remote assessment. The primary focus was on the availability of information on psychometric properties, and the secondary focus on the reporting on practical tool characteristics. The findings of this review will be used to formulate recommendations about future steps that are needed to facilitate the implementation of remote smartphone- and tablet-based tools to assess cognition.

## Methods

### Overview

This scoping review is conducted in line with a methodological framework for conducting scoping studies [32]. The reporting complies with the guidelines of the PRISMA-ScR (Preferred Reporting Items for Systematic Reviews and Meta-Analyses extension for Scoping Reviews) checklist [33], as shown in Multimedia Appendix 1. The review was not registered, and no protocol was prepared.

### Search Strategies

After several scoping searches, 4 bibliographic databases (PubMed, Embase [Elsevier], Web of Science Core Collection [Clarivate Analytics], and APA PsycINFO [EBSCO]) were searched for relevant literature from January 1, 2008 (ie, the year that the Google Play Store and Apple App Store, two of the biggest mobile app distribution platforms, were launched), to January 5, 2023. Searches were devised in collaboration with a medical information specialist (KAZ). Search terms including synonyms, closely related words, and keywords were used as free-text words (eg, “Alzheimer,” “digital,” and “cognition”). The search contained no methodological search filter that would limit results to specific study designs or languages. Duplicate studies were excluded using the R package *ASYSD* (R Foundation for Statistical Computing), an automated deduplication tool [34], which was followed by manual deduplication in EndNote (version X20.0.3; Clarivate Analytics). The full search strategy used for each database is detailed in Multimedia Appendix 2.

### Eligibility Criteria

Published journal articles were included or excluded according to the criteria outlined in Textbox 1.

**Inclusion and exclusion criteria.**

**Inclusion criteria**

- The following tool characteristics were met: (1) the digital tool aimed to measure cognition and (2) the tool was a performance-based measure.
- The study aim was to assess (one of the) psychometric properties as defined in the Consensus-Based Standards for the Selection of Health Measurement Instruments framework [29] of a digital tool to assess cognition.
- The study sample comprised (1) individuals with preclinical Alzheimer disease (AD) or individuals with AD pathological change, defined by normal cognition but presence of abnormal amyloid and tau protein biomarkers or abnormal amyloid biomarkers, respectively (henceforth, preclinical AD) [1]; or (2) individuals with mild cognitive impairment, which was diagnosed based on established clinical criteria, or it was specified that a clinical diagnosis was given in a memory clinic or (university) hospital or by a clinical or medical specialist.
- The following practical tool characteristics were met (to ensure potential for remote administration): (1) the digital tool was administered using a smartphone or tablet and (2) the digital tool was self-administered or had the potential to be self-administered, as indicated by the contextual information provided.

**Exclusion criteria**

- The studies involved self-reported diagnosis or classification solely based on AD risk factors (eg, Apolipoprotein E ε4 allele and family history of AD), or the investigated population only included individuals with dementia.
- The articles reported case studies, conference proceedings, conference abstracts, preprints, research protocols, qualitative studies, reviews, opinion papers, studies that used the digital tool as an outcome measurement instrument (eg, in randomized controlled trials), or studies that used the tool in a validation study of another instrument, or the articles were not fully available in English.
- The device of the digital tool was not specified.
- Additional equipment was required for using the digital tool (eg, stylus, digital pen, joystick, or virtual reality glasses).
- Self-administration of the digital tool was not possible, or the potential for self-administration was not indicated by contextual information.
- Automated scoring was not possible within the digital tool.

### Screening Procedures

To minimize selection bias, 2 of the review authors (RLvdB and SMvdL) independently screened titles and abstracts based on the eligibility criteria (Textbox 1) using ASReview (version 1.1) [35] with default settings (naive Bayes, term frequency–inverse document frequency, and query strategy: max). Differences in judgment were resolved via a consensus procedure through discussion and, if necessary, by consulting a third reviewer (MJK). ASReview is an open-source screening tool using manual input in combination with an active learning algorithm to screen studies based on titles and abstracts. The algorithm was trained using 7 relevant and 7 irrelevant studies, where relevant studies were manually selected and irrelevant studies were randomly provided by the model and subsequently manually labeled as irrelevant. A data-driven stopping rule strategy was followed, which has been suggested previously, and proposes to end the screening process after *n* consecutive irrelevant records are provided by the active learning algorithm [36]. No consensus exists yet on the optimal *n*, and the number varies across studies (eg, 20-500) [36-41]. Importantly, it has previously been noted that the stopping rule should aim to find the trade-off between the costs of manually labeling additional records and the costs of erroneous exclusions of relevant records by the algorithm [42]. We stopped the screening process after 100 consecutive irrelevant studies were provided by the ASReview algorithm. This number was based on an initial pilot screening round where it was observed that, if the stopping rule was set to 50 consecutive irrelevant records, relevant records continued to appear after 45 to 49 records, so the cutoff was increased to 100 to include a higher certainty margin. After screening of titles and abstracts, 4 of the review authors (RLvdB, SMvdL, MJK, and AMvG) conducted full-text screening according to the eligibility criteria (Textbox 1) using the web application Rayyan (Qatar Computing Research Institute) [43]. Each study was screened by one of the reviewers independently. Uncertainties were discussed, and if this resulted in disagreement, this was resolved through discussion, or if necessary, a fifth reviewer was consulted.

### Data Extraction and Data Synthesis

#### Overview

Two review authors (RLvdB and MJK) independently extracted data from the final set of included studies such that each study was assessed by 2 authors. Uncertainties were discussed, and if needed, a third author was consulted (SMvdL, AMvG, MvD, SAMS, and CdB). Data were extracted about practical tool characteristics, study characteristics, and psychometric properties. Practical tool characteristics and psychometric properties were extracted from the included studies and summarized at the tool level. Summarized information was calculated based on available data, and missing data were excluded. Study characteristics were extracted and reported at the study level. In case of missing information, this was reported as such. Examples of practical characteristics and psychometric properties were provided narratively.

Practical tool characteristics concerned the cognitive domains assessed (single or multiple), active or passive task completion (ie, evaluating performance during an assessment vs assessing performance in everyday life activities, respectively), administration time, testing design (ie, single or repeated assessment), and language in which the tool was examined in the included studies. Study characteristics included the study population examined, total sample size and investigative setting (on-site, eg, in the clinic, university, or day or community center, or remote, eg, at-home environment). In addition, we extracted information on the psychometric properties of the included digital tools, which we selected based on recommendations on how cognition should be measured as formulated in the “recommendation framework for evaluation of performance based cognitive tests” [16] related to construct validity and interpretability that were based on the Consensus-Based Standards for the Selection of Health Measurement Instruments methodology. The psychometric properties assessed in this review were (1) structural validity (factor analysis), (2) construct validity (clinical and biological associations and relevant clinical group differences), (3) test-retest reliability and responsiveness, and (4) interpretability (clinical meaningfulness), which are defined and explained in more detail in the following sections. Specifically, for each digital tool, we evaluated whether information on each of these 4 psychometric properties was reported (✓) or not available in the included studies. The ratings of ✓ and “not available” were assigned regardless of the method used or the quality of the reported psychometric property.

#### Structural Validity

Structural validity refers to the degree to which scores are an adequate reflection of the dimensionality that is measured (ie, underlying structure of one or multiple cognitive domains) [16,29]. As such, it is recommended to conducted a factor analysis to demonstrate adequate use of outcome scores [16]. The digital tool was given a rating of ✓ if a factor analysis was conducted.

#### Construct Validity

Construct validity refers to the extent to which outcome scores are in line with hypotheses regarding, for example, relationships to scores on other instruments or differences between relevant groups [29]. For clinical outcome assessments in early AD, it is recommended that test scores are validated against relevant clinical or biological measures [16]. We defined three subcategories for construct validity: (1) clinical association, (2) biological association, and (3) relevant group differences. The tool was assigned a rating of ✓ for clinical associations if correlations were evaluated between the digital outcome measures and traditional measures of global cognition or specific cognitive domains. The tool was given a rating of ✓ for biological associations if correlations between AD biomarkers (eg, β-amyloid and tau protein deposition), markers of neurodegeneration (eg, measures of cortical thickness), or AD biomarker group differences on the digital outcome measure were reported. The tool was given a rating of ✓ if differences between groups with different clinical status (eg, cognitively unimpaired [CU], MCI, and AD dementia) were reported.

#### Reliability and Responsiveness

Test-retest reliability refers to the extent to which cognitive performance measured using a digital tool is consistent for the same patient over time [29,44]. Given that a patient has not changed, no cognitive changes are expected for the same patient under the same conditions within a short time frame. The tool was assigned a rating of ✓ if test-retest reliability was reported.

Responsiveness refers to the sensitivity of a digital tool to detect change in cognition over time [16,29]. As such, a requirement for digital tools aiming at measuring cognitive change is that they are sensitive to it. Hence, responsiveness is an essential property of digital tools, and a validation study should be conducted on the ability to capture cognitive change in the target population [16]. Tools were assigned a rating of ✓ if responsiveness of the digital tool to cognitive change over time was reported.

#### Interpretability

Clinical meaningfulness is not considered a psychometric property but, rather, an important tool characteristic for interpretability of a (change in) score [29]. Clinical meaningfulness refers to a score (or its change) that can be interpreted as clinically relevant such that a qualitative meaning is assigned to the quantitative score [16,29]. For outcome measures in early AD, it is recommended that the scores that patients and caregivers perceive as clinically meaningful be examined [16]. We assigned the tool a rating of ✓ if it specified what (change in) score was considered clinically relevant. The tool was assigned a rating of “not available” if it did not specify what (change in) score was considered clinically relevant or if the term “clinical meaningfulness” was used but no defined meaningful score was provided.

## Results

### Search and Screening Results

**Figure 1:**
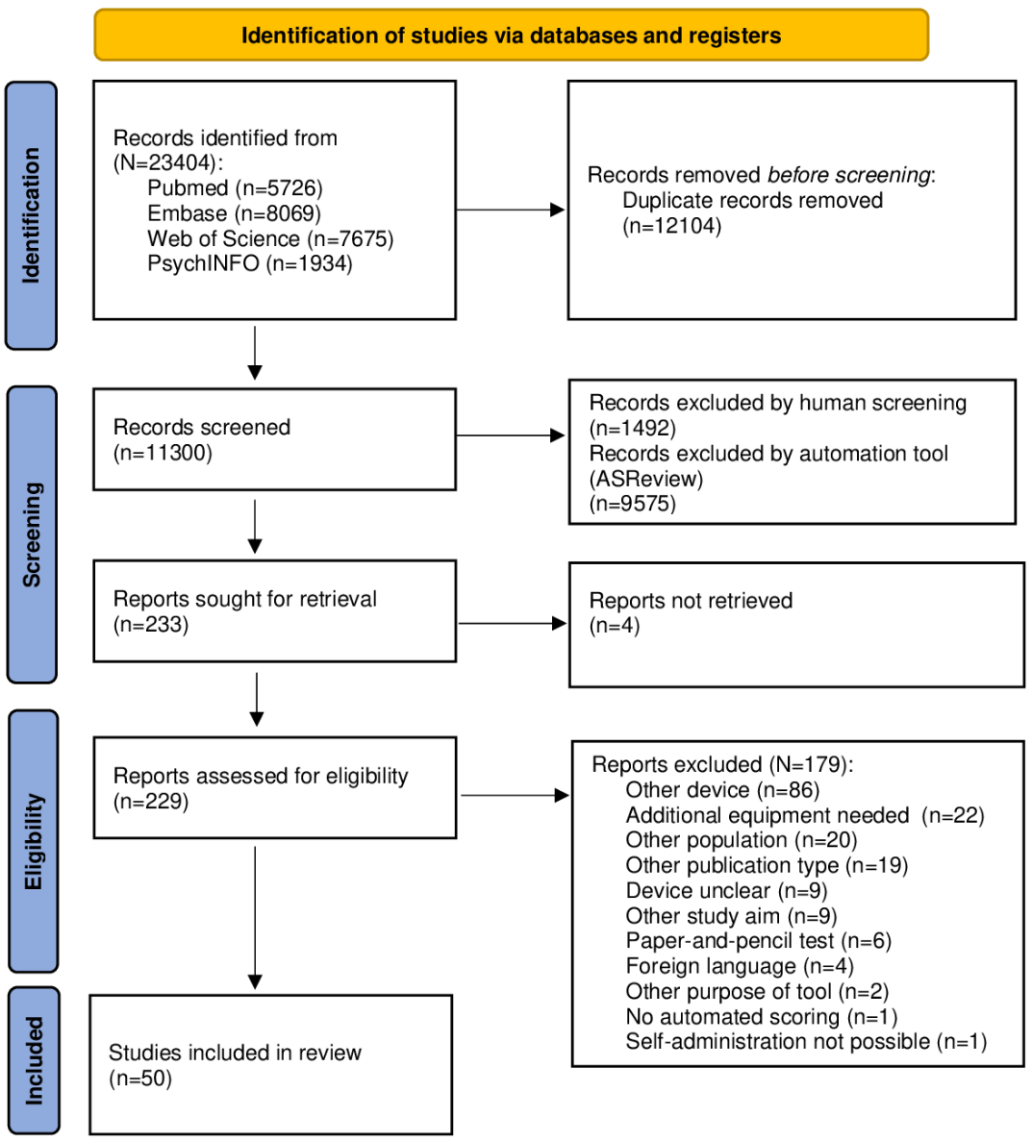
PRISMA flowchart of the search and selection procedure of the studies included in the scoping review.

The systematic literature search generated a total of 23,404 references: 5726 (24.47%) in PubMed, 8069 (34.48%; n=11,322, 48.38% with conference abstracts) in Embase, 7675 (32.79%) in Web of Science Core Collection, and 1934 (8.26%) in APA PsycINFO. After removing duplicates, of the 23,404 references, 11,300 (48.28%) remained. These references were screened based on title and abstract in ASReview. Of the 11,300 abstracts, the first reviewer (RLvdB) manually included 233 (2.06%) relevant ones and excluded 1465 (12.96%) irrelevant ones, and the second reviewer (SMvdL) manually included 234 (2.07%) relevant ones and excluded 1062 (9.4%) irrelevant ones. Within these 2 sets, there was agreement for 33.4% (156/467) of the included abstracts and 38.3% (969/2527) of the excluded abstracts, whereas 1.27% (144/11,300) of the abstracts were labeled differently (eg, included or excluded) such that, within the set of studies that were manually labeled by both reviewers, there was 88% agreement. After reaching consensus, 233 relevant studies were identified and included for further full-text screening. After full-text screening, of the 233 studies, 50 (21.5%) were included for data extraction. The PRISMA (Preferred Reporting Items for Systematic Reviews and Meta-Analyses) flowchart [45] of the search and selection process is presented in Figure 1.

### Tool and Study Characteristics

#### Practical Tool Characteristics

In total, 50 studies were included that comprised 37 tools. Although it is complex to make a binary distinction between one-to-one digitalized versions of paper-and-pencil tests and tools that are based to some extent on existing traditional paper-and-pencil tests, it was observed that digital tools used a variety of approaches. One-to-one digitalized versions of widely used traditional paper-and-pencil tests included, for example, a digital trail making task (digital Trail Making Test–Black and White) [46], digitalized word list recall (ReVeRe word list recall) [47], a computerized associative learning task (paired associate learning [PAL]) [48], and an automated story recall task (ASRT) [49,50]. The digitalization of these existing paper-and-pencil tests allowed for new variables, such as movement and reaction time measures. Other tools comprised batteries of multiple digital tests that were based on existing neuropsychological tasks (eg, the Boston Remote Assessment for Neurocognitive Health [BRANCH] [51], cCOG [52], and Electronic Cognitive Screen [53]). Other approaches involved, for example, tapping as rapidly as possible or tapping following a presented sound rhythm (JustTouch [54]), identifying nonidentical stimuli between 2 presented blocks (the tablet-based cancellation test e-CT [55]), placing virtual objects and recalling their location (Altoida [56-58]), or remembering names and occupations associated with faces (FACEmemory [59,60]). One tool, Klondike Solitaire [61,62], incorporated an “off-the-shelf” game evaluating performance on the existing digitalized game Klondike solitaire. Other methods involved tasks simulating everyday life activities, such as dosing pills in a virtual pill box (Simulation-Based Assessment of Cognition [SIMBAC] [63,64]) or grocery shopping in a virtual supermarket (Virtual Supermarket Test [VST] [65-68]). Moreover, approaches included the measurement of everyday communication skills, such as typing (nQ Medical [69] and Type of Mood [70]) and speech production (ki:e speech biomarker for cognition [SB-C] [71], Winterlight Assessment [WLA] [72], and a “tablet-based automatic assessment using speech data” [73]).

Information about practical tool characteristics is shown in Table 1. Most tools (26/37, 70%) were reported to assess multiple cognitive domains. The cognitive domains assessed varied across tools and included, for example, attention, episodic memory, executive functioning, inhibition, orientation, language, implicit learning, processing speed, or working memory [52,74-76]. A total of 30% (11/37) of the tools assessed 1 specific domain. Of those 11 tools, 6 (55%) assessed memory (BRANCH [51], PAL [48], Episodix [77], FACEmemory [59,60], Learning Over Repeated Exposures [LORE] [78], and ReVeRe word list recall [47]). In total, 8% (3/37) of the tools assessed language (speech; WLA [72], ki:e SB-C [71], and the “tablet-based automatic assessment using speech data” [73]). A total of 3% (1/37) of the tools measured executive functioning (e-CT [55]), and 3% (1/37) measured motor function (JustTouch [54]).

All but 1 digital tool (36/37, 97%) were active cognitive tasks. The passive tool (Type of Mood [70]) measured everyday typing performance. However, it should be noted that the active or passive dichotomization should be placed on a continuum where, for instance, tasks that are not commonly performed in everyday life, such as the e-CT [55] (canceling nonmatching symbols) or JustTouch [54] (rapidly tapping), might be considered more on the active side. On the other hand, tools analyzing actively elicited speech (eg, WLA [72], ki:e SB-C [71], and the “tablet-based automatic assessment using speech data” [73]) or typing a copied text or simulated text conversation (nQ Medical app [69]) might be considered more passive testing because such activities may be argued to interfere with everyday activities to a lesser extent.

**Table 1:** Practical tool characteristics.

Tool	Cognitive domain	Passive or active	Administration time	Testing design	Language examined in the included studies
Altoida [56-58]	Multiple	Active	8 min	Varied across the studies—single or repeated	English, French, Spanish, Greek, German, and Italian
ARC^a^ [22]	Multiple	Active	—^b^	Repeated	English
ASRT^c^ [49,50]	Multiple	Active	3-5 min	Repeated	English
BRANCH^d^ [51]	Memory (episodic, associative, and visual)	Active	22 min	Single	English
cCOG [52]	Multiple	Active	20 min	Single	English, Finnish, Danish, Dutch, and Italian
CompCog [74]	Multiple	Active	—	—	Portuguese
CAMCI^e^ [79]	Multiple	Active	20 min	Single	English
C3^f^ [80,81]	Multiple	Active	25-30 min	Single	English
dTMT-B&W^g^ [46]	Multiple	Active	—	Single	Korean
EC-Screen^h^ [53]	Multiple	Active	5 min	Single	Chinese
Episodix [77]	Memory (episodic)	Active	—	—	Spanish
Face-name associative memory exam (FACEmemory) [59,60]	Memory (episodic)	Active	30 min	Single	Spanish
GSCT^i^ [82]	Multiple	Active	16-48 min	Single	Swedish
Inbrain CST^j^ [83]	Multiple	Active	30 min	Single	Korean
ICA^k^ [84]	Multiple	Active	5 min	Single	Farsi and English
JustTouch [54]	Motor function	Active	1 min	Single	Japanese
ki:e SB-C^l^ [71]	Language (speech)	Active	—	—	Dutch and English
Klondike Solitaire [61,62]	Multiple	Active	—	—	Dutch (Belgian)
LORE^m^ [78]	Memory (associative)	Active	—	Repeated	English
Miro Health mobile assessment platform [85]	Multiple	Active	—	—	English
M2C2^n^ [86]	Multiple	Active	4-5 min	Repeated	English
mSTS-MCI^o^ [87]	Multiple	Active	15 min	Single	Korean
NNCT^p^ [76]	Multiple	Active	10 min	Single	—
NeuroUX [88]	Multiple	Active	11 min	Repeated	English
NIHTB-CB^q^ [75]	Multiple	Active	— “brief”	—	English
nQ Medical mobile phone app [69]	Multiple (via keystroke dynamics)	Active	—	—	English
PAL^r^ task [48]	Memory (episodic)	Active	— “brief”	Single	English
ReVeRe WLR^s^ [47]	Memory (episodic)	Active	—	—	English
SIMBAC^t^ [64]	Multiple	Active	10 min	Single	English
SMART^u^ [89]	Multiple	Active	5 min	Single	English
Tablet-based cancellation test (e-CT) [55]	Executive functioning	Active	2 min	Single	French
Type of Mood mobile app [70]	Multiple	Passive	—	Repeated	Greek
UCSF-BHA^v^ (different versions) [90-93]	Multiple	Active	10 min	Single	English and Spanish
VST^w^ (different versions) [65-68]	Multiple	Active	25-30 min	Single	Greek and Turkish
WLA^x^ [72]	Language (speech)	Active	5-10 min	Single	English
“A tablet-based cognitive assessment” [94]	Multiple	Active	10 min	Single	Taiwanese
“Tablet-based automatic assessment using speech data” [73]	Language (speech)	Active	—	—	Japanese

^a^ARC: Ambulatory Research in Cognition.

^b^Not available (ie, not described in the study).

^c^ASRT: automated story recall task.

^d^BRANCH: Boston Remote Assessment for Neurocognitive Health.

^e^CAMCI: Computer Assessment of Mild Cognitive Impairment.

^f^C3: Computerized Cognitive Composite.

^g^dTMT-B&W: digital Trail Making Test–Black and White.

^h^EC-Screen: Electronic Cognitive Screen.

^i^GSCT: Geras Solutions cognitive test.

^j^CST: Cognitive Screening Test.

^k^ICA: Integrated Cognitive Assessment.

^l^SB-C: speech biomarker for cognition.

^m^LORE: Learning Over Repeated Exposures.

^n^M2C2: Mobile Monitoring of Cognitive Change.

^o^mSTS-MCI: mobile screening test system for mild cognitive impairment.

^p^NNCT: Natural and Artificial Intelligence Health Assistant Neuro Cognitive Test.

^q^NIHTB-CB: National Institutes of Health Toolbox Cognition Battery.

^r^PAL: paired associate learning.

^s^WLR: word list recall.

^t^SIMBAC: Simulation-Based Assessment of Cognition.

^u^SMART: Survey for Memory, Attention, and Reaction Time.

^v^UCSF-BHA: University of California, San Francisco, Brain Health Assessment.

^w^VST: Virtual Supermarket Test.

^x^WLA: Winterlight Assessment.

Administration time was, on average, 13.8 (SD 10.1; range 1-32) minutes. The shortest tasks involved finger tapping; symbol cancelation; story recall; and a short battery of a symbol-matching, color-shape-memory, and location-memory task (JustTouch [54], e-CT [55], ASRT [49,50], and Mobile Monitoring of Cognitive Change [86]). The tools with the longest administration time covered multiple tasks assessing different cognitive domains (Geras Solutions cognitive test [82] and Inbrain Cognitive Screening Test [CST] [83]) or learning face-name-occupation associations and memorizing these after a 20-minute delay (FACEmemory [59,60]). For 38% (14/37) of the tools, the administration time was unspecified, and for 24% (9/37) of the tools, the testing design (ie, single or repeated testing) was not reported. Most tools (21/37, 57%) had a single-testing design, whereas 16% (6/37) were incorporated into a multiday protocol, and for 3% (1/37) of the tools (Altoida [56-58]), the testing design differed across studies. In the repeated-testing designs, (1) participants completed short cognitive tasks over several days (Altoida [56,58], Ambulatory Research in Cognition [ARC] [22], ASRT [49,50], NeuroUX [88], and Mobile Monitoring of Cognitive Change [86]), (2) tasks were assessed monthly for 1 year (LORE [78]), or (3) passive monitoring comprised a longer period (ie, 6 months; Type of Mood [70]).

Most tools (31/37, 84%) were tested in a single language, which was primarily English. A total of 11% (4/37) of the tools were tested in 2 languages (ie, VST [65-68] in Greek and Turkish; University of California, San Francisco, Brain Health Assessment [UCSF-BHA] [90-93] in English and Spanish; ki:e SB-C [71] in English and Dutch; and Integrated Cognitive Assessment [84] in English and Farsi). In total, 3% (1/37) of the tools were assessed in 5 languages (ie, cCOG [52] in English, Finnish, Danish, Dutch, and Italian), and 3% (1/37) were assessed in 6 languages (ie, Altoida [56-58] in English, French, Spanish, Greek, German, and Italian). For 3% (1/37) of the tools (Natural and Artificial Intelligence Health Assistant Neuro Cognitive Test [76]), the language was not specified.

#### Study Characteristics

Table 2 provides a summary of the characteristics of the included studies that examined a smartphone- or tablet-based cognitive tool in individuals with MCI or preclinical AD. Most tools (29/37, 78%) were assessed in a single study. A total of 14% (5/37) of the tools were examined in 4% (2/50) of the studies (ASRT [49,50], Computerized Cognitive Composite [C3] [80,81], FACEmemory [59,60], Klondike Solitaire [61,62], and SIMBAC [63,64]). One tool (Altoida [56-58]) was assessed in 6% (3/50) of the included studies, and 5% (2/37) of the tools (VST [65-68] and UCSF-BHA [90-93]) were examined in 8% (4/50) of the studies. Most of the included studies (36/50, 72%) had a cross-sectional design, whereas 28% (14/50) had a longitudinal design. These longitudinal studies assessed 32% (12/37) of the digital tools.

The mean total sample size in the included studies was 295.4 (SD 621.6), with a range of 12 to 4486 individuals. Most studies (41/50, 82%) examined the digital tool in individuals with MCI but not in individuals with preclinical AD, 10% (5/50) of the studies included both individuals with MCI and individuals with preclinical AD, and 8% (4/50) of the studies included individuals with preclinical AD but not individuals with MCI. Summarizing these studies at an individual tool level, most tools (29/37, 78%) were examined in individuals with MCI but not in individuals with preclinical AD. In total, 8% (3/37) of the digital tools (ARC [22], C3 [80,81], and LORE [78]) were investigated in individuals with preclinical AD but not in individuals with MCI. A total of 14% (5/37) of the tools (ASRT [49,50], BRANCH [51], FACEmemory [59,60], PAL [48], and UCSF-BHA [90-93]) were examined in both individuals with preclinical AD and individuals with MCI. Thus, most tools were investigated in individuals with MCI (34/37, 92%), and fewer tools were examined in individuals with preclinical AD (8/37, 22%). A total of 54% (27/50) of the studies, covering 59% (22/37) of the tools, additionally included populations other than individuals with preclinical AD or MCI, such as individuals with dementia.

In 48% (24/50) of the studies the digital tools were examined on-site. In 22% (11/50) of the studies, the psychometric properties of the tool were investigated in a remote setting, and in 16% (8/50) of the studies, these properties were examined in both an on-site and remote setting. In 14% (7/50) of the studies, the investigative setting was not described.

**Table 2:** Study characteristics^a^.

Study	Tool	Study design	Examined populations	Total sample size	Investigative setting
Rai et al [57], 2020	Altoida	Longitudinal	CU^b^ and MCI^c^	496	On-site and remote
Meier et al [56], 2021	Altoida	Longitudinal	CU, MCI, and dementia	525	Remote
Seixas et al [58], 2022	Altoida	Longitudinal	CU, MCI, and dementia	576	On-site and remote
Nicosia et al [22], 2023	ARC^d^	Longitudinal	CU, preclinical AD^e^, and dementia	290	Remote
Skirrow et al [50], 2022	ASRT^f^	Longitudinal	CU, MCI, and dementia	151	On-site and remote
Fristed et al [49], 2022	ASRT	Cross-sectional	CU, preclinical AD, MCI, and dementia	115	Remote
Papp et al [51], 2021	BRANCH^g^	Cross-sectional	CU, preclinical AD, and MCI	234	On-site and remote
Rhodius-Meester et al [52], 2020	cCOG	Longitudinal	CU, MCI, and dementia	495	On-site and remote
Hartle et al [74], 2022	CompCog	Cross-sectional	CU and MCI	52	On-site
Saxton et al [79], 2009	CAMCI^h^	Cross-sectional	CU and MCI	524	On-site
Papp et al [80], 2021	C3^i^	Cross-sectional	CU and preclinical AD	4486	On-site
Jutten et al [81], 2021	C3	Longitudinal	CU and preclinical AD	114	On-site and remote
Simfukwe et al [46], 2022	dTMT-B&W^j^	Cross-sectional	CU and MCI	44	—^k^
Chan et al [53], 2020	EC-Screen^l^	Cross-sectional	CU, MCI, and dementia	243	On-site
Valladares-Rodriguez et al [77], 2018	Episodix	Cross-sectional	CU, MCI, and dementia	64	On-site
Alegret et al [59], 2020	FACEmemory	Cross-sectional	CU, preclinical AD, and MCI	276	On-site
Alegret et al [60], 2022	FACEmemory	Cross-sectional	MCI	94	On-site
Bloniecki et al [82], 2021	GSCT^m^	Cross-sectional	SCD^n^, MCI, and dementia	98	On-site
Chin et al [83], 2020	Inbrain CST^o^	Cross-sectional	SCD, MCI, and dementia	577	On-site
Kalafatis et al [84], 2021	ICA^p^	Cross-sectional	CU, MCI, and dementia	230	On-site or remote with presence of researcher
Suzumura et al [54], 2018	JustTouch	Cross-sectional	CU, MCI, and dementia	94	—
Tröger et al [71], 2022	ki:e SB-C^q^	Longitudinal	CU, SCD, and MCI	159	On-site and remote
Gielis et al [61], 2021	Klondike Solitaire	Cross-sectional	CU and MCI	46	Remote with presence of researcher
Gielis et al [62], 2021	Klondike Solitaire	Cross-sectional	CU and MCI	46	Remote with presence of researcher
Samaroo et al [78], 2020	LORE^r^	Longitudinal	CU and preclinical AD	94	Remote
Sloane et al [85], 2022	Miro Health platform	Cross-sectional	CU, MCI, and other	174	—
Cerino et al [86], 2021	M2C2^s^	Cross-sectional	CU and MCI	311	Remote
Park et al [87], 2018	mSTS-MCI^t^	Cross-sectional	CU and MCI	177	On-site
Oliva and Losa [76], 2022	NNCT^u^	Cross-sectional	CU, MCI, and dementia	147	—
Moore et al [88], 2022	NeuroUX	Longitudinal	CU and MCI	94	Remote
Ma et al [75], 2021	NIHTB-CB^v^	Cross-sectional	CU, MCI, dementia, and other	411	On-site
Holmes et al [69], 2022	nQ Medical app	Cross-sectional	CU and MCI	77	On-site
Pettigrew et al [48], 2022	PAL^w^	Cross-sectional	CU, preclinical AD, and MCI	73	—
Morrison et al [47], 2018	WLR^x^	Cross-sectional	CU and MCI	121	On-site
Ip et al [64], 2017	SIMBAC^y^	Cross-sectional	CU, MCI, dementia, and other	173	On-site
Rapp et al [63], 2018	SIMBAC	Cross-sectional	CU, MCI, and dementia	161	On-site
Dorociak et al [89], 2021	SMART^z^	Cross-sectional	CU and MCI	69	Remote
Wu et al [55], 2017	e-CT	Cross-sectional	CU, MCI, and dementia	325	On-site
Ntracha et al [70], 2020	Type of Mood	Longitudinal	SCD and MCI	23	Remote
Possin et al [93], 2018	UCSF-BHA^aa^	Cross-sectional	CU, SCD, MCI, and dementia	347	On-site
Tsoy et al [91], 2020	UCSF-BHA	Longitudinal	Preclinical AD, CU, MCI, and dementia	850	On-site
Tsoy et al [92], 2021	UCSF-BHA	Cross-sectional	MCI and dementia	140	On-site
Rodríguez-Salgado et al [90], 2021	UCSF-BHA	Cross-sectional	CU, MCI, and dementia	146	On-site
Zygouris et al [66], 2015	VST^ab^	Cross-sectional	CU and MCI	55	On-site
Zygouris et al [68], 2017	VST	Longitudinal	CU and MCI	12	Remote
Zygouris et al [67], 2020	VST	Cross-sectional	SCD and MCI	95	On-site
Eraslan Boz et al [65], 2020	VST	Cross-sectional	CU and MCI	89	—
Robin et al [72], 2021	WLA^ac^	Longitudinal	CU, MCI, and dementia	50	On-site
Huang et al [94], 2019	“A tablet-based cognitive assessment”	Cross-sectional	CU, MCI, and dementia	120	—
Yamada et al [73], 2021	“Tablet-based automatic assessment using speech data”	Cross-sectional	CU and MCI	76	On-site


^a^Terminology for individuals who were cognitively unimpaired or individuals with subjective cognitive decline varied across the included studies (ie, cognitively unimpaired comprised cognitively unimpaired, cognitively healthy, cognitively normal, cognitively intact, [healthy] controls, healthy older adults, no impairment, and no diagnosis of dementia, and subjective cognitive decline comprised subjective memory complaints, subjective cognitive impairment, and normal with concerns). For individuals with mild cognitive impairment, no distinction was made between amnestic and nonamnestic mild cognitive impairment or between mild cognitive impairment due to Alzheimer disease or to other causes. For individuals with dementia, no distinction was made among mild, moderate, or severe dementia or between Alzheimer disease dementia and non–Alzheimer disease dementia. Sample sizes include the total number of participants included for analyses in the studies.

^b^CU: cognitively unimpaired.

^c^MCI: mild cognitive impairment.

^d^ARC: Ambulatory Research in Cognition.

^e^AD: Alzheimer disease.

^f^ASRT: automated story recall task.

^g^BRANCH: Boston Remote Assessment for Neurocognitive Health.

^h^CAMCI: Computer Assessment of Mild Cognitive Impairment.

^i^C3: Computerized Cognitive Composite.

^j^dTMT-B&W: digital Trail Making Test–Black and White.

^k^Not available (ie, not described in the study).

^l^EC-Screen: Electronic Cognitive Screen.

^m^GSCT: Geras Solutions cognitive test.

^n^SCD: subjective cognitive decline.

^o^CST: Cognitive Screening Test.

^p^ICA: Integrated Cognitive Assessment.

^q^SB-C: speech biomarker for cognition.

^r^LORE: Learning Over Repeated Exposures.

^s^M2C2: Mobile Monitoring of Cognitive Change.

^t^mSTS-MCI: mobile screening test system for MCI.

^u^NNCT: Natural and Artificial Intelligence Health Assistant Neuro Cognitive Test.

^v^NIHTB-CB: National Institutes of Health Toolbox Cognition Battery.

^w^PAL: paired associate learning.

^x^WLR: word list recall.

^y^SIMBAC: Simulation-Based Assessment of Cognition.

^z^SMART: Survey for Memory, Attention, and Reaction Time.

^aa^UCSF-BHA: University of California, San Francisco, Brain Health Assessment.

^ab^VST: Virtual Supermarket Test.

^ac^WLA: Winterlight Assessment.

### Psychometric Properties

#### Overview

Table 3 provides a summary at the tool level on the reporting on psychometric properties in the included studies.

**Table 3:** Reported psychometric properties for the digital cognitive tools^a^.

Tool	Structural validity: factor analysis	Construct validity				Reliability and responsiveness			Interpretability: clinical meaningfulness
		Clinical associations	Biological associations	Relevant group differences	Test-retest reliability		Responsiveness		
Altoida [56-58]	—^b^	—	✓^c^	✓	—		✓	—	
ARC^d^ [22]	—	✓	✓	✓	✓		—	—	
ASRT^e^ [49,50]	—	✓	✓	✓	—		—	—	
BRANCH^f^ [51]	—	✓	✓	✓	✓		—	—	
cCOG [52]	—	✓	—	✓	✓		—	—	
CompCog [74]	—	—	—	✓	—		—	—	
CAMCI^g^ [79]	—	—	—	✓	✓		—	—	
C3^h^ [80,81]	—	✓	✓	✓	✓		✓	—	
dTMT-B&W^i^ [46]	—	✓	—	✓	—		—	—	
EC-Screen^j^ [53]	—	—	—	✓	—		—	—	
Episodix [77]	—	—	—	✓	—		—	—	
FACEmemory [59,60]	—	✓	✓	✓	—		—	—	
GSCT^k^ [82]	—	✓	—	✓	—		—	—	
Inbrain CST^l^ [83]	✓	✓	✓	✓	✓		—	—	
ICA^m^ [84]	—	✓	—	✓	—		—	—	
JustTouch [54]	—	✓	—	✓	—		—	—	
ki:e SB-C^n^ [71]	—	✓	—	✓	✓		✓	—	
Klondike Solitaire [61,62]	—	—	—	✓	—		—	—	
LORE^o^ [78]	—	—	✓	✓	—		✓	—	
Miro Health platform [85]	—	✓	—	✓	✓		—	—	
M2C2^p^ [86]	—	—	—	✓	—		—	—	
mSTS-MCI^q^ [87]	—	✓	—	✓	✓		—	—	
NNCT^r^ [76]	—	✓	—	✓	—		—	—	
NeuroUX [88]	—	✓	—	✓	—		—	—	
NIHTB-CB^s^ [75]	✓	—	—	—	—		—	—	
nQ Medical app [69]	—	✓	—	—	—		—	—	
PAL^t^ [48]	—	—	✓	✓	—		—	—	
WLR^u^ [47]	—	✓	—	✓	—		—	—	
SIMBAC^v^ [63,64]	—	✓	—	✓	✓		—	—	
SMART^w^ [89]	—	✓	—	✓	✓		—	—	
e-CT [55]	—	—	—	✓	—		—	—	
Type of Mood [70]	—	✓	—	✓	—		—	—	
UCSF-BHA^x^ (different versions) [90-93]	—	✓	✓	✓	—		✓	—	
VST^y^ (different versions) [65-68]	—	✓	—	✓	—		—	—	
WLA^z^ [72]	—	✓	—	✓	✓		✓	—	
“A tablet-based cognitive assessment” [94]	✓	✓	—	✓	—		—	—	
“Tablet-based automatic assessment using speech data” [73]	—	—	—	✓	—		—	—	

^a^The available information varied across psychometric properties—factor analysis information was provided for 8% (3/37) of the tools, clinical association information was provided for 68% (25/37) of the tools, biological association information was provided for 27% (10/37) of the tools, relevant group difference information was provided for 95% (35/37) of the tools, test-retest reliability information was provided for 32% (12/37) of the tools, responsiveness information was provided for 16% (6/37) of the tools, and clinical meaningfulness information was provided for 0% of the tools.

^b^Not available (ie, the psychometric property was not examined in the study).

^c^Indicates that the psychometric property was examined in the study.

^d^ARC: Ambulatory Research in Cognition.

^e^ASRT: automated story recall task.

^f^BRANCH: Boston Remote Assessment for Neurocognitive Health.

^g^CAMCI: Computer Assessment of Mild Cognitive Impairment.

^h^C3: Computerized Cognitive Composite.

^i^dTMT-B&W: digital Trail Making Test–Black and White.

^j^EC-Screen: Electronic Cognitive Screen.

^k^GSCT: Geras Solutions cognitive test.

^l^CST: Cognitive Screening Test.

^m^ICA: Integrated Cognitive Assessment.

^n^SB-C: speech biomarker for cognition.

^o^LORE: Learning Over Repeated Exposures.

^p^M2C2: Mobile Monitoring of Cognitive Change.

^q^mSTS-MCI: mobile screening test system for mild cognitive impairment.

^r^NNCT: Natural and Artificial Intelligence Health Assistant Neuro Cognitive Test.

^s^NIHTB-CB: National Institutes of Health Toolbox Cognition Battery.

^t^PAL: paired associate learning.

^u^WLR: word list recall.

^v^SIMBAC: Simulation-Based Assessment of Cognition.

^w^SMART: Survey for Memory, Attention, and Reaction Time.

^x^UCSF-BHA: University of California, San Francisco, Brain Health Assessment.

^y^VST: Virtual Supermarket Test.

^z^WLA: Winterlight Assessment.

#### Structural Validity

For a minority of digital tools (3/37, 8%; Inbrain CST [83], National Institutes of Health Toolbox Cognition Battery [NIHTB-CB] [75], and the tablet-based cognitive test battery [94]), a factor analysis on outcome variables was conducted to explore the underlying structure of the construct. The factor structure was examined using a multiple-factor analysis, both an exploratory and confirmatory factor analysis, or a confirmatory factor analysis, respectively. For 5% (2/37) of the tools, (structural) validity was formally assessed (NIHTB-CB [75] and the tablet-based cognitive test battery [94]), whereas for other tools, limited details were provided with regard to methodology and model fit (Inbrain CST [83]).

#### Construct Validity

For most tools (36/37, 97%), construct validity was assessed with regard to at least one aspect (ie, clinical or biological associations or relevant group differences). For the tool that was not assessed regarding construct validity (NIHTB-CB [75]), the study focused rather on its structural validity. Specifically, a clinical association was examined for 68% (25/37) of the tools. Existing clinical tests that digital tools were correlated with were, for example, the Mini-Mental State Examination, Montreal Cognitive Assessment, Rey Auditory Verbal Learning Test, or Preclinical Alzheimer’s Cognitive Composite (PACC) [52,66,71,76,80,82,94]. Correlation coefficients differed largely between tools as well as between scores within tools. To illustrate, depending on the subscale and clinical test, correlations ranged from 0.174 to 0.735 for the Natural and Artificial Intelligence Health Assistant Neuro Cognitive Test [76], from −0.02 to −0.57 for ARC [22], and from −0.382 to 0.617 for BRANCH [51].

Biological validation was evaluated for a minority of the tools (10/37, 27%). Specifically, 11% (4/37) of the tools (ASRT [49], BRANCH [51], C3 [80,81], and LORE [78]) were validated against AD biomarkers (ie, amyloid and tau protein). In total, 3% (1/37) of the tools were validated against a neuroanatomical correlate of cortical thickness (Inbrain CST [83]), and 14% (5/37) of the tools (Altoida [58], ARC [22], FACEmemory [59,60], PAL [48], and UCSF-BHA [91-93]) were validated against both AD biomarkers and neuroanatomical correlates (eg, cortical, subcortical, cerebral, and hippocampal volumes). All 22% (8/37) of the tools that were assessed in a population of individuals with preclinical AD were validated against biological measures. The methodology used to assess biological associations was (1) calculating correlations with continuous biological measures, (2) using regression analyses to assess differences between biomarker groups, or (3) conducting receiver operating characteristic analyses to predict biomarker status. Reported correlation coefficients ranged between and within tools depending on the outcome measure and biological correlate, as illustrated, for example, by correlations ranging from −0.23 to 0.29 for the ARC [22] composite score or correlations ranging from −0.306 to 0.219 for BRANCH [51] subtasks. Similarly, in receiver operating characteristic analyses, the reported area under the curve (AUC) values for predicting amyloid positivity varied between and within tools, as illustrated by AUC values of 0.752 for UCSF-BHA [92] (combination of outcome scores) and AUC values ranging from 0.43 to 0.92 for ASRT [49] depending on the subtask, the sample (full sample, CU, or MCI), and whether data transcription was manual or automatic.

Relevant clinical group differences in digital outcome scores were examined for most tools (35/37, 95%). The tool’s ability to distinguish between clinical groups was reported for different clinical groups. For example, group differences were assessed for (1) large-contrast groups (CU vs dementia, eg, cCOG [52], e-CT [55], UCSF-BHA [90], and JustTouch [54]) and (2) smaller-contrast groups (eg, CompCog [74], digital Trail Making Test–Black and White [46], cCOG [52], e-CT [55], UCSF-BHA [90], and JustTouch [54] for CU vs MCI and JustTouch [54] for MCI vs dementia).

#### Reliability and Responsiveness

Test-retest reliability was examined for a minority (12/37, 32%) of the digital tools. The methods used to assess test-retest reliability were (1) intraclass correlation coefficients (ICCs; ARC [22]; Inbrain CST [83]; Miro Health platform [85]; the mobile screening test system for MCI [87]; SIMBAC [63]; and Survey for Memory, Attention, and Reaction Time [89]), (2) correlations (ie, Pearson correlations: BRANCH [51], cCOG [52], Computer Assessment of Mild Cognitive Impairment [79], SIMBAC [64], and WLA [72]; Spearman rank partial correlations: ki:e SB-C [71]), or (3) linear models to assess performance differences between test and retest assessments (C3 [80]). The settings in which test-retest reliabilities were assessed differed across the studies that were on-site (C3 [80], Computer Assessment of Mild Cognitive Impairment [79], and Inbrain CST [83]), remote (WLA [72]), or both on-site and remote (cCOG [52]). The test-retest reliability differed across tools and was largely dependent on the outcome score. It is inherent to digital tools that they generate large amounts of outcome scores, for each of which reliability features could be reported. This results in a range from low to high reliability coefficients within a tool, as reflected in ICCs that ranged from 0.49 to 0.91 for Inbrain CST [83] and Pearson correlations that ranged from 0.24 to 0.82 for cCOG [52] or from 0.38 to 0.83 for WLA [72]. For other tools, high reliability was consistently found for multiple scores, such as for the mobile screening test system for MCI [87], where ICCs ranged from 0.97 to 0.98.

For 5% (2/37) of the tools, both test-retest reliability and responsiveness were assessed (ki:e SB-C [71] and WLA [72]). Responsiveness was reported for 16% (6/37) of the tools (Altoida [56], C3 [81], ki:e SB-C [71], LORE [78], UCSF-BHA [91], and WLA [72]). The methodologies used to assess changes in digital scores over time were linear mixed-effects models (C3 [81], LORE [78], UCSF-BHA [91], and WLA [72]), a computation of a change score (ki:e SB-C [71]), or a longitudinal intraindividual variability metric that was compared between diagnostic groups (Altoida [56]). If a digital tool generated multiple outcome scores, the responsiveness could vary depending on the specific score. For example, for the WLA [72], rates of decline in 4 aggregate scores were assessed, where 1 of those scores was found to decline more rapidly for individuals with MCI or early AD than for individuals with Montreal Cognitive Assessment scores above the threshold for cognitive impairment (≥26). For another tool (C3 [81]), 8 subtask scores and 1 composite score were generated, with most of these scores showing responsiveness to change over time, and change in 4 of these scores was reported to be associated with amyloid or tau protein burden. For 2 tools (C3 [81] and LORE [78]), improvement over time was demonstrated, where individuals without cognitive impairment with AD pathology showed less steep learning curves than controls, indicating that diminished learning curves may also be a promising indicator of cognitive decline.

#### Interpretability

Clinically meaningful (changes in) scores were not defined for any of the digital tools (0%). However, for 11% (4/37) of the tools, information on clinical meaningfulness or related aspects was argued to have been demonstrated, whereas defined meaningful scores were not provided such that clinical meaningfulness was not assessed according to its definition as provided in the Methods section. C3 [80] was demonstrated to be associated with AD biomarkers and the PACC such that the authors concluded that this tool captures meaningful cognitive decrements. The ki:e SB-C [71] tool was associated with scores on the Clinical Dementia Rating scale and differed both between diagnostic groups and between those who cognitively declined versus those who did not, which the authors interpreted as support for the tool’s clinical relevance. For LORE [78], the authors argued that diminished learning curves on the digital measurement were meaningful, whereas further research is required to quantify its clinical meaningfulness. Hence, although the term “clinically meaningful” was used in these studies, clinical meaningfulness was not examined according to its definition, which presupposes a defined meaningful score [16,29].

## Discussion

### Principal Findings

In this scoping review, we identified 50 studies covering 37 different smartphone- and tablet-based tools to assess cognition in individuals with preclinical AD or MCI. These numbers indicate that multiple smartphone- and tablet-based cognitive tools are currently being developed, whereas only a limited number of validation studies in this target population have been conducted for individual tools. Specifically, except for construct validity, most psychometric properties (ie, structural validity, reliability, responsiveness, and clinical meaningfulness) have not been extensively assessed to date. Thus, considering the foundations of measurement, as reflected in the psychometric properties, these tools are still in the early stages of validation. Moreover, it was observed that practical characteristics such as administration time or the investigative setting were not consistently reported. Although the initial reporting on the psychometric properties of smartphone- and tablet-based tools to assess cognition in early AD support their potential, further and careful validation of their psychometric properties, as well as clear information on practical characteristics, is essential to facilitate implementation.

Practical tool characteristics may be argued to be important toward considering the tool’s use in clinical practice, supported by previous observations that, for health care professionals, time efficiency would facilitate the use of digital tools [26]. We highlight that, for a significant number of tools, information was not reported regarding administration time (14/37, 38%), the testing design (single or repeated; 9/37, 24%), or the investigative setting (on-site or remote; 7/37, 19%). This is concerning as information on the setting is crucial to interpret whether the psychometric properties assessed are applicable for on-site or remote use of a tool and for a single (eg, annual) or repeated (eg, daily or monthly) measurement. Especially given that the advantages of digital tools include their time-efficient administration and potential for remote and highly frequent administration, we recommend that such characteristics be clearly reported.

The generation of high-dimensional data is inherent to digital tools that assess cognition. While this may be regarded as an advantage, we observed that it also poses challenges to the overall evaluation of the quality of a tool and complicates one-to-one comparisons between tools. For instance, it remains challenging to decide which scores should be evaluated and compared, which raises questions such as whether the psychometric properties of single scores or aggregated scores should be considered when appraising the quality of the digital tool and whether the tool’s quality is determined by the outcome score that holds the lowest or highest psychometric quality. These challenges of high-dimensional datasets were specifically observed in the psychometric properties of construct validity and test-retest reliability, which widely ranged for the number of generated scores of a single tool. We recommend that the promising large amounts of generated data be reduced carefully by selecting those outcome scores that hold high psychometric quality, which we consider a crucial step to develop digital cognitive tools that provide clinically useful outcome scores. Another observation was that different methodologies were used between tools to determine their psychometric properties. This highlights the need for more uniformity regarding psychometric evaluation, which would facilitate the comparison of digital tools regarding their psychometric quality, which is needed to enable the end user to select the most appropriate tool for its context of use.

To standardize the process of clinical evaluation of digital tools, we propose a more widespread use of operating procedures, such as the FDA guidance on software as a medical device [95]. This guideline recognizes the importance of psychometric property evaluation and recommends 3 steps. The first step concerns clinical association, where the digital outcome should be demonstrated to be associated with the targeted clinical condition (ie, clinical decline as a result of AD). The second evaluation step regards analytical validation of the software, which is out of scope for this review. The third step involves clinical validation, where the digital outcome should be demonstrated to be reliable and clinically meaningful with regard to its intended purpose. The importance of provided information on psychometric properties is further emphasized by the previously reported notion that clear information on psychometric properties would facilitate the use of digital tools in clinical practice [26]. To this end, we will discuss the reporting on individual psychometric properties in the following paragraphs.

A first psychometric property that could be argued to be placed at the first step of the clinical evaluation process regards the conduct of factor analyses to confirm that outcome scores adequately reflect the dimensionality that is measured (ie, the underlying structure of one or multiple cognitive domains). In this review, we observed that a factor analysis on outcome scores was conducted for a limited number of digital tools (3/37, 8%) to demonstrate the underlying structure of the measured construct. This is concerning, and it is advisable for future validation studies to increasingly focus on conducting factor analyses to ensure the digital tool’s structural validity.

Another psychometric property regarding demonstrating clinical associations is construct validity, which was reported for most digital tools (36/37, 97%). Correlation levels between generated digital scores and scores on gold-standard measures of cognition ranged significantly both within and between tools, similarly to previous findings regarding self-administered computerized cognitive assessments [31]. It has been highlighted that, as currently used cognitive tests may not be suitable for early AD stages, validation against these “gold standard” tests may be particularly relevant in more progressed stages, whereas validation against biological measures of AD may be more relevant in preclinical stages [16,17,20]. Recently, proposed revisions to the diagnostic criteria for AD have been published, with the common denominator that preclinical AD concerns a stage characterized by the absence of symptoms [96,97]. Hence, to demonstrate an association with the disease in this stage, biological validation is needed instead of clinical validation, for which no gold-standard test exists at this stage. This importance of biological validation in the preclinical AD stage was reflected in our observation that validation against biological measures was reported for fewer tools (10/37, 27%) and all tools that were assessed in the preclinical stage were validated against biological measures. Still, most tools (36/37, 97%) were validated against clinical measures, including tools that were examined in preclinical AD. In the absence of a true gold standard for preclinical AD, it remains concerning that novel sensitive tests for preclinical populations are nonetheless validated against existing tests with limited sensitivity. As suggested previously, predictive validity might be considered as an alternative “copper standard” [16]. As we did not evaluate the reporting on predictive validity, further research should examine the ability of smartphone- and tablet-based tools for use in early AD stages to predict future cognitive decline or progression to MCI or dementia.

The third clinical evaluation step, clinical validation, involves demonstrating reliability. Clinical trials examining potential intervention effects inherently require repeated assessments over time, and to accurately interpret change in cognition over time, the test-retest reliability of an outcome measure should be ensured in the target population. The test-retest reliability was examined for a limited number of digital tools included in this review (12/37, 32%). As we selected studies that focused on the MCI and preclinical stages, it may be the case that test-retest reliability was assessed in studies focusing on other populations, including, for example, individuals with dementia. However, as test-retest reliability may differ across populations, it is important that this psychometric property is assessed in the target population. The importance of ensuring the tool’s reliability may be emphasized even more in the context of self-administered digital tools that may be more susceptible to noise than supervised paper-and-pencil tests. For the tools that had available information on the test-retest reliability, the reliability levels ranged substantially, largely depending on the outcome score of a tool (eg, total score or single scores), in line with previous observations [31]. This makes it highly challenging to determine the overall reliability of a specific tool and stresses the importance of careful selection of outcome scores. We recommend that the test-retest reliability should be thoroughly considered before selecting outcome scores for further validation steps. In addition, as the large absence of information on this psychometric property may hinder the implementation of digital tools, validation studies should increasingly focus on evaluating test-retest reliability. Alternatively, although we note that the independent validation studies are preferable, we recommend that the evaluation of test-retest reliability also be incorporated into clinical trial designs where, for example, the natural course of a digital outcome should be attested first before investigating its change after the intervention.

To determine whether a digital cognitive tool is sensitive to measure potential treatment effects or cognitive changes over time for monitoring purposes, information on the tool’s responsiveness is essential. Responsiveness was reported for a small number of tools (6/37, 16%) using different methodologies, and findings varied within the outcome scores of a single tool. Interestingly, of these 6 tools, 2 (33%) showed that less steep improvement was associated with preclinical AD [78,81]. Thus, although it may be expected that declining performance would be indicative of cognitive change, these new digital repeated testing approaches showed that diminished learning curves may also serve as an indicator of cognitive change in individuals with preclinical AD. Our finding that responsiveness was evaluated to a lesser extent may be explained by the fact that it requires a more effortful, longitudinal study design. In addition, this low number of tools for which responsiveness was reported may reflect the relatively young character of the research field involving digital tools to assess cognition in early AD stages, which may indicate that these tools are not ready for clinical implementation yet and warrant further validation. Accordingly, it is recommended that future validation studies focus on examining the responsiveness of digital outcome scores to change over time in the target population as an essential step toward clinical implementation.

Another aspect of the third clinical validation step regards clinical meaningfulness. To enable clinical interpretation of digital scores and clinically relevant change, the clinical meaningfulness of scores and their change should be determined. However, no consensus has been established yet on what score should be considered clinically meaningful [98]. The importance of clinically meaningful end points has also been stated by the US Food and Drug Administration draft guidance from March 2024 [99], where it has been recognized that, in early AD stages, it may be challenging to demonstrate clinical meaningfulness due to subtle cognitive changes and the absence of overt cognitive impairment. With the lack of a clear definition of clinical meaningfulness in early AD stages, this quality characteristic may be easily overlooked in the validation of digital cognitive tools, as reflected in our observation that clinically meaningful scores were not defined for any of the identified digital tools. However, it should be noted that, for some digital outcome scores, it was argued that their meaningfulness was supported by associations with AD biomarkers and clinical measures (PACC or Clinical Dementia Rating), as well as by scores or learning curves that differed between diagnostic groups [71,78,80]. Such definitions of clinical meaningfulness related to clinical, biological, and discriminative validity seem to refer to the relevance of the measure rather than the magnitude of the score or change in score that demonstrated clinical meaningfulness [100]. Hence, there is a pressing need for a clear definition of clinical meaningfulness, and it has been suggested that this definition should be developed by incorporating perspectives of various stakeholders (eg, patients, clinicians, and regulatory bodies) [98]. In addition, clinical meaningfulness may differ across patients, for which digital tools may offer an opportunity to determine personalized end points. Moreover, norm scores or cutoff scores may be considered toward establishing clinical meaningfulness. Thus, it is recommended that, within the field concerning cognitive assessment in early-stage AD in general, attention should be paid to reaching consensus on the definition of clinical meaningfulness of digital outcome scores to enable clinically useful interpretation of digital outcomes in future validation studies.

The major strength of this review was that we focused on the preclinical AD and MCI stages. These predementia stages offer the greatest potential for benefit from intervention strategies as well as having the highest need and offering the greatest feasibility in the context of novel digital remote assessments. Another strength was that we used a systematic search to identify validation studies of digital tools to assess cognition, which allowed for a large selection of studies. In addition, as our scope covered a range of psychometric properties that were selected based on previous recommendations, as well as practical tool characteristics that enabled the identification of an “information gap” in both areas, this allowed for the provision of a clear overview on what information is missing and, thus, what should be focused on to support the implementation of digital tools.

This review has a number of limitations. First, although we used a broad and systematic search strategy to minimize the chance of missing studies, the identified studies relied on specific search terms such that potentially relevant studies might have been excluded. For example, studies were not identified within the search if keywords related to the digital aspect of tools, such as “digital,” “computerized,” “smartphone,” or “tablet,” were not used in the title or abstract. Second, we did not conduct quality assessments of the included studies, which may have potentially biased our overall conclusions. Third, because this is a rapidly developing field, new digital cognitive assessments will be continuously emerging, highlighting the need for ongoing evaluation. Therefore, our aim was not to provide an elaborate overview of currently available tools but rather identify possible information gaps on reported psychometric properties in the landscape of digital cognitive tools. In addition, our scope was limited to tools that had the potential for remote assessment. The evaluation of this characteristic was not based on a strict criterion but rather on the contextual information provided indicating the suitability for self-administration that implies the potential for remote administration, which may, thus, be slightly subjective. We decided not to formulate a stricter eligibility criterion for this remote design as, for most tools, it was not explicitly stated whether the tool was intended for remote assessment. As this is in fact important information, it is recommended that future validation studies provide clear information on the intended context of the digital tool. Moreover, as we focused on validation studies, we did not include development studies, nor did we assess additional information on the tools’ websites on the tool characteristics, such as administration time, intended use, or available languages. Therefore, we emphasize that the provided overview of such characteristics may not adequately reflect all practical information or languages available to date but rather provide an overview of all available information as reported in validation studies targeting a population of individuals with preclinical AD or MCI. However, it is crucial to include such information in scientific publications for end users to decide which tools are most appropriate for their context of use and to evaluate the quality of digital cognitive tools in future systematic reviews [101]. Furthermore, we did not evaluate the quality of the methods used to assess the psychometric properties, and it is subject to discussion whether any method used was appropriate. For instance, to demonstrate structural validity, confirmatory factor analyses are argued to be the preferred method over exploratory factor analyses [102-104]. Similarly, to evaluate the quality of the tool’s construct validity, it is of relevance whether the clinical or biological correlations or group differences assessed are in the hypothesized direction, and for evaluating responsiveness, the time frame (eg, days, weeks, or months) should be considered. We provided a first step to identify the reporting on psychometric properties regardless of their quality, but future research should focus on the quality of the methodology used to determine psychometric properties.

### Conclusions

Our results indicate that the landscape of smartphone- and tablet-based cognitive tools for early AD could currently be placed at the first step in the validation process, as described in the Food and Drug Administration guidance on software as a medical device [95]. Following this guidance, it is recommended that, after establishing a valid clinical association, digital tools should be further validated with regard to their context of use. For the development of novel tools, we recommend clearly stating the digital tool’s intended use to determine the prioritized validation steps with regard to the most relevant psychometric properties. For instance, if a tool would be used for monitoring purposes, demonstrating responsiveness is crucial. For digital screening tools, sensitivity should be demonstrated, whereas for digital diagnostic tools, the specificity for a clinical condition should be validated. As we identified multiple gaps in the available information on crucial psychometric properties, which halts the clinical validation of these tools, this stresses the need for more attention on psychometric quality, where the selection of outcome scores should be considered carefully and reported consistently. As a next step, future studies should determine the psychometric quality of currently developed digital tools for use in early AD stages. Ensuring psychometrically high-quality outcome scores must be a central theme in the development of smartphone- and tablet-based tools that assess cognition in individuals with preclinical AD and MCI. Further clinical validation tailored to the context of use will pave the way for the implementation of digital cognitive tools.

## Appendix

Multimedia Appendix 1 PRISMA-ScR checklist.

Multimedia Appendix 2 Search strategies.

## Abbreviations

AD: Alzheimer disease
ARC: Ambulatory Research in Cognition
ASRT: automated story recall task
AUC: area under the curve
BRANCH: Boston Remote Assessment for Neurocognitive Health
C3: Computerized Cognitive Composite
CST: Cognitive Screening Test
CU: cognitively unimpaired
ICC: intraclass correlation coefficient
LORE: Learning Over Repeated Exposures
MCI: mild cognitive impairment
NIHTB-CB: National Institutes of Health Toolbox Cognition Battery
PACC: Preclinical Alzheimer’s Cognitive Composite
PAL: paired associate learning
PRISMA: Preferred Reporting Items for Systematic Reviews and Meta-Analyses
PRISMA-ScR: Preferred Reporting Items for Systematic Reviews and Meta-Analyses extension for Scoping Reviews
SB-C: speech biomarker for cognition
SIMBAC: Simulation-Based Assessment of Cognition
UCSF-BHA: University of California, San Francisco, Brain Health Assessment
VST: Virtual Supermarket Test
WLA: Winterlight Assessment
