# Supplementary material for: Smartphone- and Tablet-Based Tools to Assess Cognition in Individuals With Preclinical Alzheimer Disease and Mild Cognitive Impairment: Scoping Review
Source: J Med Internet Res. 2025 May 27;27:e65297. doi: 10.2196/65297 (PMC12152440; doi:10.2196/65297)
Supplement: Multimedia Appendix 2 [file jmir_v27i1e65297_app2.docx]

**Supplementary Table 2.** Search histories

(A). Search history PUBMED January 5, 2023

| **Search** | **PubMed Query January 5, 2023** | **Results** |
| --- | --- | --- |
| 6 | #5 AND (2008:3000[pdat]) | 5726 |
| 5 | #1 AND #2 AND (#3 OR #4) | 6640 |
| 4 | "Controlled Oral Word Association Test"[tiab] OR "COWAT"[tiab] OR "Rey Complex Figure Test"[tiab] OR "Rey-Osterrieth Complex Figure"[tiab] OR "Clock Test"[tiab] OR "Digit span"[tiab] OR "Letter Digit Substitution"[tiab] OR "Montreal Cognitive Assessment"[tiab] OR "MOCA"[tiab] OR "Mini Mental State Examination"[tiab] OR "MMSE"[tiab] OR "Clinical Dementia Rating"[tiab] OR "Dementia Rating Scale"[tiab] OR "verbal fluency"[tiab] OR "phonemic fluency"[tiab] OR "letter fluency"[tiab] OR "category fluency"[tiab] OR "animal fluency"[tiab] OR "trail making test"[tiab] OR "15 words test"[tiab] OR "Rey’s Auditory Verbal Learning"[tiab] OR "stroop test"[tiab] OR "Visual Object and Space Perception Battery"[tiab] OR "Number location"[tiab] OR "Fragmented letters"[tiab] OR "Visual Association test"[tiab] OR "Dutch Adult Reading test"[tiab] OR "Boston naming test"[tiab] OR "Apraxia test van Heugten"[tiab] OR "Wechsler Memory Scale visual representation"[tiab] OR "Rivermead Behavioural Memory test"[tiab] | 43767 |
| 3 | "Memory"[Mesh] OR "Executive Function"[Mesh] OR "Attention"[Mesh:NoExp] OR "Cognitive Dysfunction"[Mesh:NoExp] OR "Cognition"[Mesh:NoExp] OR "Neuropsychological Tests"[Mesh] OR "Mental Status and Dementia Tests"[Mesh] OR "Cognitive Reserve"[Mesh] OR "cognition"[tiab] OR "cognitive dysfunct*"[tiab] OR "neurocognitive dysfunct*"[tiab] OR "neuropsychological dysfunct*"[tiab] OR "memory dysfunct*"[tiab] OR "attention dysfunct*"[tiab] OR "executive function*"[tiab] OR "executive dysfunction*"[tiab] OR "learning dysfunct*"[tiab] OR "cognitive impair*"[tiab] OR "cognitive reserve"[tiab] OR "neurocognitive impair*"[tiab] OR "neuropsychological impair*"[tiab] OR "memory impair*"[tiab] OR "attention impair*"[tiab] OR "executive impair*"[tiab] OR "processing speed impair*"[tiab] OR "learning impair*"[tiab] OR "cognitive deficit*"[tiab] OR "neurocognitive deficit*"[tiab] OR "neuropsychological deficit*"[tiab] OR "memory deficit*"[tiab] OR "attention deficit*"[tiab] OR "executive deficit*"[tiab] OR "processing speed deficit*"[tiab] OR "learning deficit*"[tiab] OR "cognitive failur*"[tiab] OR "cognitive function*"[tiab] OR "cognitive declin*"[tiab] OR "neurocognitive loss*"[tiab] OR "cognitive loss*"[tiab] OR visuospat*[tiab] OR visuoconstruct*[tiab] OR "processing speed"[tiab] OR "perceptual speed"[tiab] OR "spatial navigation"[tiab] OR "cognitive battery"[tiab] OR "cognitive progression"[tiab] OR "cognitive deteriorat*"[tiab] OR "cognitive dysfunction"[tiab] OR "cognitive chang*"[tiab] OR "orientation"[tiab] OR "working memory"[tiab] OR "spatial memory"[tiab] OR "episodic memory"[tiab] OR "executive control"[tiab] OR "executive function*"[tiab] OR "cognitive control"[tiab] OR language[tiab] OR speech[tiab] OR linguistic[tiab] OR fluency[tiab] | 1056717 |
| 2 | Computers, handheld"[Mesh] OR "Mobile Applications"[Mesh] OR "Internet"[Mesh]" OR "mobile health*"[tiab] OR "mhealth*"[tiab] OR "m health*"[tiab] OR "ehealth*"[tiab] OR "e-health*"[tiab] OR "digital health"[tiab] OR "app"[tiab] OR "apps"[tiab] OR "smartphone*"[tiab] OR "phone application*"[tiab] OR "telephone application*"[tiab] OR "mobile application*"[tiab] OR "mobile technolog*"[tiab] OR "health technolog*"[tiab] OR "health application*"[tiab] OR "internet*"[tiab] OR "world wide web"[tiab] OR "webportal*"[tiab] OR "web portal*"[tiab] OR "patient portal*"[tiab] OR "ipad"[tiab] OR "ipads"[tiab] OR "perrsonal digital assistant*"[tiab] OR "patient monitoring"[tiab] OR "multimedia"[tiab] OR "Mhapps"[tiab] OR "iphone*"[tiab] OR "android"[tiab] OR "game*"[tiab] OR "gaming"[tiab] OR "gamification"[tiab] OR "exergam*"[tiab] OR "serious gam*"[tiab] OR "computer*"[tiab] OR "digital*"[tiab] OR "remote"[tiab] OR "self assessment*"[tiab] OR "self administ*"[tiab] OR "home assessment*"[tiab] OR "web based"[tiab] OR "application based"[tiab] OR "app based"[tiab] OR "tablet"[tiab] OR "iPadOS"[tiab] OR "iOS"[tiab] OR "touchscreen"[tiab] OR "ecological momentary assessment"[tiab] OR "google play store"[tiab] OR "software as medical device"[tiab] | 934068 |
| 1 | "Alzheimer Disease"[Mesh] OR "alzheimer*"[tiab] OR "mild cognitive impairment"[tiab] OR "subjective cognitive decline"[tiab] OR "subjective memory impairment"[tiab] OR "subjective memory complain*"[tiab] OR "ATN"[tiab] OR "subjective cognitive impairment"[tiab] OR "subjective cognitive complain*"[tiab] OR amyloid* [tiab] | 263374 |

(B) Search history EMBASE.COM January 5, 2023

| **Search** | **Embase Query – January 5, 2023** | **Results** |
| --- | --- | --- |
| #7 | #6 NOT ‘conference abstract’/it | 8068 |
| #6 | #5 AND (2008:py OR 2009:py OR 2010:py OR 2011:py OR 2012:py OR 2013:py OR 2014:py OR 2015:py OR 2016:py OR 2017:py OR 2018:py OR 2019:py OR 2020:py OR 2021:py OR 2022:py OR 2023:py) | 11322 |
| #5 | #1 AND #2 AND (#3 OR #4) | 12495 |
| #4 | 'controlled oral word association test':ti,ab,kw OR 'cowat':ti,ab,kw OR 'rey complex figure test':ti,ab,kw OR 'rey-osterrieth complex figure':ti,ab,kw OR 'clock test':ti,ab,kw OR 'digit span':ti,ab,kw OR 'letter digit substitution':ti,ab,kw OR 'montreal cognitive assessment':ti,ab,kw OR 'moca':ti,ab,kw OR 'mini mental state examination':ti,ab,kw OR 'mmse':ti,ab,kw OR 'clinical dementia rating':ti,ab,kw OR 'dementia rating scale':ti,ab,kw OR 'verbal fluency':ti,ab,kw OR 'phonemic fluency':ti,ab,kw OR 'letter fluency':ti,ab,kw OR 'category fluency':ti,ab,kw OR 'animal fluency':ti,ab,kw OR 'trail making test':ti,ab,kw OR '15 words test':ti,ab,kw OR 'rey auditory verbal learning':ti,ab,kw OR 'stroop test':ti,ab,kw OR 'visual object and space perception battery':ti,ab,kw OR 'number location':ti,ab,kw OR 'fragmented letters':ti,ab,kw OR 'visual association test':ti,ab,kw OR 'dutch adult reading test':ti,ab,kw OR 'boston naming test':ti,ab,kw OR 'apraxia test van heugten':ti,ab,kw OR 'wechsler memory scale visual representation':ti,ab,kw OR 'rivermead behavioural memory test':ti,ab,kw | 74172 |
| #3 | 'mild cognitive impairment'/exp OR 'memory'/exp OR 'attention'/exp OR 'cognitive reserve'/exp OR 'cognition'/de OR 'executive function'/exp OR 'cognitive defect'/de OR 'neuropsychological test'/exp OR 'dementia assessment'/exp OR 'mental capacity'/de OR 'orientation'/exp OR 'cognition':ti,ab,kw OR 'cognitive dysfunct*':ti,ab,kw OR 'neurocognitive dysfunct*':ti,ab,kw OR 'neuropsychological dysfunct*':ti,ab,kw OR 'memory dysfunct*':ti,ab,kw OR 'attention dysfunct*':ti,ab,kw OR 'executive dysfunction*':ti,ab,kw OR 'learning dysfunct*':ti,ab,kw OR 'cognitive impair*':ti,ab,kw OR 'cognitive reserve':ti,ab,kw OR 'neurocognitive impair*':ti,ab,kw OR 'neuropsychological impair*':ti,ab,kw OR 'memory impair*':ti,ab,kw OR 'attention impair*':ti,ab,kw OR 'executive impair*':ti,ab,kw OR 'processing speed impair*':ti,ab,kw OR 'learning impair*':ti,ab,kw OR 'cognitive deficit*':ti,ab,kw OR 'neurocognitive deficit*':ti,ab,kw OR 'neuropsychological deficit*':ti,ab,kw OR 'memory deficit*':ti,ab,kw OR 'attention deficit*':ti,ab,kw OR 'executive deficit*':ti,ab,kw OR 'processing speed deficit*':ti,ab,kw OR 'learning deficit*':ti,ab,kw OR 'cognitive failur*':ti,ab,kw OR 'cognitive function*':ti,ab,kw OR 'cognitive declin*':ti,ab,kw OR 'neurocognitive loss*':ti,ab,kw OR 'cognitive loss*':ti,ab,kw OR visuospat*:ti,ab,kw OR visuoconstruct*:ti,ab,kw OR 'processing speed':ti,ab,kw OR 'perceptual speed':ti,ab,kw OR 'spatial navigation':ti,ab,kw OR 'cognitive battery':ti,ab,kw OR 'cognitive progression':ti,ab,kw OR 'cognitive deteriorat*':ti,ab,kw OR 'cognitive dysfunction':ti,ab,kw OR 'cognitive chang*':ti,ab,kw OR 'orientation':ti,ab,kw OR 'working memory':ti,ab,kw OR 'spatial memory':ti,ab,kw OR 'episodic memory':ti,ab,kw OR 'executive control':ti,ab,kw OR 'executive function*':ti,ab,kw OR 'cognitive control':ti,ab,kw OR language:ti,ab,kw OR speech:ti,ab,kw OR linguistic:ti,ab,kw OR fluency:ti,ab,kw | 1726955 |
| #2 | 'telehealth'/de OR 'telemedicine'/de OR 'telemonitoring'/exp OR 'internet'/exp OR 'computer interface'/exp OR 'multimedia'/exp OR 'mobile phone'/exp OR 'microcomputer'/exp OR 'mobile application'/exp OR 'mobile health*':ti,ab,kw OR 'mhealth*':ti,ab,kw OR 'm health*':ti,ab,kw OR 'ehealth*':ti,ab,kw OR 'e-health*':ti,ab,kw OR 'digital health':ti,ab,kw OR 'app':ti,ab,kw OR 'apps':ti,ab,kw OR 'smartphone*':ti,ab,kw OR 'phone application*':ti,ab,kw OR 'telephone application*':ti,ab,kw OR 'mobile application*':ti,ab,kw OR 'mobile technolog*':ti,ab,kw OR 'health technolog*':ti,ab,kw OR 'health application*':ti,ab,kw OR 'internet*':ti,ab,kw OR 'world wide web':ti,ab,kw OR 'webportal*':ti,ab,kw OR 'web portal*':ti,ab,kw OR 'patient portal*':ti,ab,kw OR 'ipad':ti,ab,kw OR 'ipads':ti,ab,kw OR 'perrsonal digital assistant*':ti,ab,kw OR 'patient monitoring':ti,ab,kw OR 'multimedia':ti,ab,kw OR 'mhapps':ti,ab,kw OR 'iphone*':ti,ab,kw OR 'android':ti,ab,kw OR 'game*':ti,ab,kw OR 'gaming':ti,ab,kw OR 'gamification':ti,ab,kw OR 'exergam*':ti,ab,kw OR 'serious gam*':ti,ab,kw OR 'computer*':ti,ab,kw OR 'digital*':ti,ab,kw OR 'remote':ti,ab,kw OR 'self assessment*':ti,ab,kw OR 'self administ*':ti,ab,kw OR 'home assessment*':ti,ab,kw OR 'web based':ti,ab,kw OR 'application based':ti,ab,kw OR 'app based':ti,ab,kw OR 'tablet':ti,ab,kw OR 'ipados':ti,ab,kw OR 'ios':ti,ab,kw OR 'touchscreen':ti,ab,kw OR 'ecological momentary assessment':ti,ab,kw OR 'google play store':ti,ab,kw OR 'software as medical device':ti,ab,kw | 1343464 |
| #1 | 'alzheimer disease'/exp OR 'alzheimer*':ti,ab,kw OR 'mild cognitive impairment':ti,ab,kw OR 'subjective cognitive decline':ti,ab,kw OR 'subjective memory impairment*':ti,ab,kw OR 'subjective memory complain*':ti,ab,kw OR 'atn':ti,ab,kw OR 'mild cognitive complain*':ti,ab,kw OR 'subjective cognitive impairment':ti,ab,kw OR 'subjective cognitive complain*':ti,ab,kw | 308395 |

(C) Search history EBSCO/APA PSYCHINFO January 5, 2023

| **Search** | **PsycInfo Query – January 5, 2023** | **Results** |
| --- | --- | --- |
| S6 | S5 AND Limiters – Publication Year: 2008-2023 | 1934 |
| S5 | S1 AND S2 AND (S3 OR S4) | 2484 |
| S4 | TM “Controlled Oral Word Association Test” OR “COWAT” OR “Rey Complex Figure Test” OR Rey-Osterrieth Complex Figure” OR “Clock Test” OR “Digit span” OR “Letter Digit Substitution” OR “Montreal Cognitive Assessment” OR “MOCA” OR “Mini Mental State Examination” OR “MMSE” OR “Clinical Dementia Rating” OR “Dementia Rating Scale” OR “verbal fluency” OR “phonemic fluency” OR “letter fluency” OR “semantic fluency” OR “category fluency” OR “animal fluency” OR “trail making test” OR “15 words test” OR “Rey’s Auditory Verbal Learning” OR “stroop test” OR “Visual Object and Space Perception Battery” OR “Number location” OR “Fragmented letters” OR “Visual Association test” OR “Dutch Adult Reading test” OR “Boston naming test” OR “Apraxia test van Heugten” OR “Wechsler Memory Scale visual representation” OR “Rivermead Behavioural Memory test” | 4541 |
| S3 | DE ("Memory" OR "Associative Memory" OR "Autobiographical Memory" OR "Collective Memory" OR "Early Memories" OR "Eidetic Imagery" OR "Episodic Memory" OR "Explicit Memory" OR "False Memory" OR "Forgetting" OR "Implicit Memory" OR "Long Term Memory" OR "Memory Consolidation" OR "Memory Decay" OR "Memory Trace" OR "Prospective Memory" OR "Reminiscence" OR "Retrospective Memory" OR "Short Term Memory" OR "Spatial Memory" OR "Tip of the Tongue Phenomenon" OR "Visual Memory" OR "Visuospatial Memory" OR "Spatial Learning" OR "Spatial Navigation" OR "Spatial Ability" OR "Visuospatial Ability" OR "Spatial Orientation (Perception)" OR "Wayfinding" OR "Executive Function" OR "Cognitive Ability" OR "Cognitive Processes" OR "Cognitive Impairment" OR "Awareness" OR "Cognition" OR "Cognitions" OR "Cognitive Processing Speed" OR "Cognitive Reserve" OR "Concentration" OR "Executive Function" OR "Human Channel Capacity" OR "Human Information Storage" OR "Neuropsychological Assessment" OR "Mini Mental State Examination" OR "Cognitive Assessment" OR "Attention" OR "Attentional Capture" OR "Distraction" OR "Divided Attention" OR "Focused Attention" OR "Monitoring" OR "Selective Attention" OR "Sustained Attention" OR "Vigilance" OR "Visual Attention" OR "Attention Span") OR TI (“cognition” OR “cognitive dysfunct*” OR “neurocognitive dysfunct*” OR “neuropsychological dysfunct*” OR “memory dysfunct*” OR “attention dysfunct*” OR “executive function*” OR “executive dysfunction*” OR “learning dysfunct*” OR “cognitive impair*” OR “cognitive reserve” OR “neurocognitive impair*” OR “neuropsychological impair*” OR “memory impair*” OR “attention impair*” OR “executive impair*” OR “processing speed impair*” OR “learning impair*” OR “cognitive deficit*” OR “neurocognitive deficit*” OR “neuropsychological deficit*” OR “memory deficit*” OR “attention deficit*” OR “executive deficit*” OR “processing speed deficit*” OR “learning deficit*” OR “cognitive failur*” OR “cognitive function*” OR “cognitive declin*” OR “neurocognitive loss*” OR “cognitive loss*” OR visuospat* OR visuoconstruct* OR “processing speed” OR “perceptual speed” OR “spatial navigation” OR “cognitive battery” OR “cognitive progression” OR “cognitive deteriorat*” OR “cognitive dysfunction” OR “cognitive chang*” OR “orientation” OR “working memory” OR “spatial memory” OR “episodic memory” OR “executive control” OR “executive function*” OR “cognitive control” OR language OR speech OR linguistic OR fluency) OR AB (“cognition” OR “cognitive dysfunct*” OR “neurocognitive dysfunct*” OR “neuropsychological dysfunct*” OR “memory dysfunct*” OR “attention dysfunct*” OR “executive function*” OR “executive dysfunction*” OR “learning dysfunct*” OR “cognitive impair*” OR “cognitive reserve” OR “neurocognitive impair*” OR “neuropsychological impair*” OR “memory impair*” OR “attention impair*” OR “executive impair*” OR “processing speed impair*” OR “learning impair*” OR “cognitive deficit*” OR “neurocognitive deficit*” OR “neuropsychological deficit*” OR “memory deficit*” OR “attention deficit*” OR “executive deficit*” OR “processing speed deficit*” OR “learning deficit*” OR “cognitive failur*” OR “cognitive function*” OR “cognitive declin*” OR “neurocognitive loss*” OR “cognitive loss*” OR visuospat* OR visuoconstruct* OR “processing speed” OR “perceptual speed” OR “spatial navigation” OR “cognitive battery” OR “cognitive progression” OR “cognitive deteriorat*” OR “cognitive dysfunction” OR “cognitive chang*” OR “orientation” OR “working memory” OR “spatial memory” OR “episodic memory” OR “executive control” OR “executive function*” OR “cognitive control” OR language OR speech OR linguistic OR fluency) OR KW (“cognition” OR “cognitive dysfunct*” OR “neurocognitive dysfunct*” OR “neuropsychological dysfunct*” OR “memory dysfunct*” OR “attention dysfunct*” OR “executive function*” OR “executive dysfunction*” OR “learning dysfunct*” OR “cognitive impair*” OR “cognitive reserve” OR “neurocognitive impair*” OR “neuropsychological impair*” OR “memory impair*” OR “attention impair*” OR “executive impair*” OR “processing speed impair*” OR “learning impair*” OR “cognitive deficit*” OR “neurocognitive deficit*” OR “neuropsychological deficit*” OR “memory deficit*” OR “attention deficit*” OR “executive deficit*” OR “processing speed deficit*” OR “learning deficit*” OR “cognitive failur*” OR “cognitive function*” OR “cognitive declin*” OR “neurocognitive loss*” OR “cognitive loss*” OR visuospat* OR visuoconstruct* OR “processing speed” OR “perceptual speed” OR “spatial navigation” OR “cognitive battery” OR “cognitive progression” OR “cognitive deteriorat*” OR “cognitive dysfunction” OR “cognitive chang*” OR “orientation” OR “working memory” OR “spatial memory” OR “episodic memory” OR “executive control” OR “executive function*” OR “cognitive control” OR language OR speech OR linguistic OR fluency) | 963040 |
| S2 | (DE "Telemedicine" OR DE "Internet" DE "Computer Mediated Communication" OR DE "Telecommunications Media" OR DE "Cellular Phones" OR DE "Computer Applications" OR DE "Multimedia" OR DE "Microcomputers" OR DE "Mobile Devices" OR DE "Social Media" OR DE "Online Social Networks" OR DE "Text Messaging") OR TI (“mobile health*” OR “mhealth*” OR “m health*” OR “ehealth*” OR “e-health*” OR “digital health” OR “app” OR “apps” OR “smartphone*” OR “phone application*” OR “telephone application*” OR “mobile application*” OR “mobile technolog*” OR “health technolog*” OR “health application*” OR “internet*” OR “world wide web” OR “webportal*” OR “web portal*” OR “patient portal*” OR “ipad” OR “ipads” OR “perrsonal digital assistant*” OR “patient monitoring” OR “multimedia” OR “mhapps” OR “iphone*” OR “android” OR “game*” OR “gaming” OR “gamification” OR “exergam*” OR “serious gam*” OR “computer*” OR “digital*” OR “remote” OR “self assessment*” OR “self administ*” OR “home assessment*” OR “web based” OR “application based” OR “app based” OR “tablet” OR “ipados” OR “ios” OR “touchscreen” OR “ecological momentary assessment” OR “google play store” OR “software as medical device”) OR AB (“mobile health*” OR “mhealth*” OR “m health*” OR “ehealth*” OR “e-health*” OR “digital health” OR “app” OR “apps” OR “smartphone*” OR “phone application*” OR “telephone application*” OR “mobile application*” OR “mobile technolog*” OR “health technolog*” OR “health application*” OR “internet*” OR “world wide web” OR “webportal*” OR “web portal*” OR “patient portal*” OR “ipad” OR “ipads” OR “perrsonal digital assistant*” OR “patient monitoring” OR “multimedia” OR “mhapps” OR “iphone*” OR “android” OR “game*” OR “gaming” OR “gamification” OR “exergam*” OR “serious gam*” OR “computer*” OR “digital*” OR “remote” OR “self assessment*” OR “self administ*” OR “home assessment*” OR “web based” OR “application based” OR “app based” OR “tablet” OR “ipados” OR “ios” OR “touchscreen” OR “ecological momentary assessment” OR “google play store” OR “software as medical device”) OR KW (“mobile health*” OR “mhealth*” OR “m health*” OR “ehealth*” OR “e-health*” OR “digital health” OR “app” OR “apps” OR “smartphone*” OR “phone application*” OR “telephone application*” OR “mobile application*” OR “mobile technolog*” OR “health technolog*” OR “health application*” OR “internet*” OR “world wide web” OR “webportal*” OR “web portal*” OR “patient portal*” OR “ipad” OR “ipads” OR “perrsonal digital assistant*” OR “patient monitoring” OR “multimedia” OR “mhapps” OR “iphone*” OR “android” OR “game*” OR “gaming” OR “gamification” OR “exergam*” OR “serious gam*” OR “computer*” OR “digital*” OR “remote” OR “self assessment*” OR “self administ*” OR “home assessment*” OR “web based” OR “application based” OR “app based” OR “tablet” OR “ipados” OR “ios” OR “touchscreen” OR “ecological momentary assessment” OR “google play store” OR “software as medical device”) | 314273 |
| S1 | DE ("Alzheimer's Disease" OR “Beta Amyloid”) OR TI (“alzheimer*” OR “mild cognitive impairment” OR “subjective cognitive decline” OR “subjective memory impairment” OR “subjective memory complain*” OR “ATN”) OR AB (“alzheimer*” OR “mild cognitive impairment” OR “subjective cognitive decline” OR “subjective memory impairment” OR “subjective memory complain*” OR “ATN”) OR KW (“alzheimer*” OR “mild cognitive impairment” OR “subjective cognitive decline” OR “subjective memory impairment” OR “subjective memory complain*” OR “ATN”) | 77118 |

(D) Search history Clarivate analytics/WEB OF SCIENCE core collection January 5, 2023

| **Search** | **Web of Science Query – January 5, 2023** | **Results** |
| --- | --- | --- |
| 1 | TS=(“alzheimer*” OR “mild cognitive impairment” OR “subjective cognitive decline” OR “subjective memory impairment” OR “subjective memory complain*” OR “ATN”) | 294556 |
| 2 | TS=(“mobile health*” OR “mhealth*” OR “m health*” OR “ehealth*” OR “e-health*” OR “digital health” OR “app” OR “apps” OR “smartphone*” OR “phone application*” OR “telephone application*” OR “mobile application*” OR “mobile technolog*” OR “health technolog*” OR “health application*” OR “internet*” OR “world wide web” OR “webportal*” OR “web portal*” OR “patient portal*” OR “ipad” OR “ipads” OR “perrsonal digital assistant*” OR “patient monitoring” OR “multimedia” OR “mhapps” OR “iphone*” OR “android” OR “game*” OR “gaming” OR “gamification” OR “exergam*” OR “serious gam*” OR “computer*” OR “digital*” OR “remote” OR “self assessment*” OR “self administ*” OR “home assessment*” OR “web based” OR “application based” OR “app based” OR “tablet” OR “ipados” OR “ios” OR “touchscreen” OR “ecological momentary assessment” OR “google play store” OR “software as medical device”) | 2332957 |
| 3 | TS=(“cognition” OR “cognitive dysfunct*” OR “neurocognitive dysfunct*” OR “neuropsychological dysfunct*” OR “memory dysfunct*” OR “attention dysfunct*” OR “executive function*” OR “executive dysfunction*” OR “learning dysfunct*” OR “cognitive impair*” OR “cognitive reserve” OR “neurocognitive impair*” OR “neuropsychological impair*” OR “memory impair*” OR “attention impair*” OR “executive impair*” OR “processing speed impair*” OR “learning impair*” OR “cognitive deficit*” OR “neurocognitive deficit*” OR “neuropsychological deficit*” OR “memory deficit*” OR “attention deficit*” OR “executive deficit*” OR “processing speed deficit*” OR “learning deficit*” OR “cognitive failur*” OR “cognitive function*” OR “cognitive declin*” OR “neurocognitive loss*” OR “cognitive loss*” OR visuospat* OR visuoconstruct* OR “processing speed” OR “perceptual speed” OR “spatial navigation” OR “cognitive battery” OR “cognitive progression” OR “cognitive deteriorat*” OR “cognitive dysfunction” OR “cognitive chang*” OR “orientation” OR “working memory” OR “spatial memory” OR “episodic memory” OR “executive control” OR “executive function*” OR “cognitive control” OR language OR speech OR linguistic OR fluency) | 1882227 |
| 4 | TS=(“controlled oral word association test” OR “cowat” OR “rey complex figure test” OR “rey-osterrieth complex figure” OR “clock test” OR “digit span” OR “letter digit substitution” OR “montreal cognitive assessment” OR “moca” OR “mini mental state examination” OR “mmse” OR “clinical dementia rating” OR “dementia rating scale” OR “verbal fluency” OR “phonemic fluency” OR “letter fluency” OR “category fluency” OR “animal fluency” OR “trail making test” OR “15 words test” OR “rey auditory verbal learning” OR “stroop test” OR “visual object and space perception battery” OR “number location” OR “fragmented letters” OR “visual association test” OR “dutch adult reading test” OR “boston naming test” OR “apraxia test van heugten” OR “wechsler memory scale visual representation” OR “rivermead behavioural memory test”) | 53631 |
| 5 | #1 AND #2 AND (#3 OR #4) | 8796 |
| 6 | #1 AND #2 AND (#3 OR #4) and 2008 or 2009 or 2010 or 2011 or 2012 or 2013 or 2014 or 2015 or 2016 or 2017 or 2018 or 2019 or 2020 or 2021 or 2022 or 2023 (Publication Years) | 7675 |
